# Prediction of Perinatal and Neurodevelopmental Outcomes in Newborns with a Birth Weight below the 3rd Percentile: Performance of Two International Curves – Prospective Cohort from a Brazilian City

**DOI:** 10.1055/s-0043-1770131

**Published:** 2023-06-20

**Authors:** Marcos Masaru Okido, Ricardo de Carvalho Cavalli, Viviane Cunha Cardoso, Alessandra Cristina Marcolin

**Affiliations:** 1 Department of Obstetrics and Gynecology, University of São Paulo, Ribeirão Preto, Brazil; 2 Department of Puericulture and Pediatrics, University of São Paulo, Ribeirão Preto, Brazil

**Keywords:** fetal growth retardation, birth weight, neurodevelopmental disorders, retardo do crescimento fetal, peso ao nascer, transtornos do neurodesenvolvimento

## Abstract

**Objectives** To evaluate the performance of Intergrowth-21 st (INT) and Fetal Medicine Foundation (FMF) curves in predicting perinatal and neurodevelopmental outcomes in newborns weighing below the 3rd percentile.

**Methods** Pregnant women with a single fetus aged less than 20 weeks from a general population in non-hospital health units were included. Their children were evaluated at birth and in the second or third years of life. Newborns (NB) had their weight percentiles calculated for both curves. Sensitivity, specificity, positive (PPV) and negative predictive value (NPV), and area under the ROC curve (ROC-AUC) for perinatal outcomes and neurodevelopmental delay were calculated using birth weight < 3rd percentile as the cutoff.

**Results** A total of 967 children were evaluated. Gestational age at birth was 39.3 (±3.6) weeks and birth weight was 3,215.0 (±588.0) g. INT and FMF classified 19 (2.4%) and 49 (5.7%) newborns below the 3rd percentile, respectively. The prevalence of preterm birth, tracheal intubation >24 hours in the first three months of life, 5th minute Apgar <7, admission to a neonatal care unit (NICU admission), cesarean section rate, and the neurodevelopmental delay was 9.3%, 3.3%, 1.3%, 5.9%, 38.9%, and 7.3% respectively. In general, the 3rd percentile of both curves showed low sensitivity and PPV and high specificity and NPV. The 3rd percentile of FMF showed superior sensitivity for preterm birth, NICU admission, and cesarean section rate. INT was more specific for all outcomes and presented a higher PPV for the neurodevelopmental delay. However, except for a slight difference in the prediction of preterm birth in favor of INT, the ROC curves showed no differences in the prediction of perinatal and neurodevelopmental outcomes.

**Conclusion** Birth weight below the 3rd percentile according to INT or FMF alone was insufficient for a good diagnostic performance of perinatal and neurodevelopmental outcomes. The analyzes performed could not show that one curve is better than the other in our population. INT may have an advantage in resource contingency scenarios as it discriminates fewer NB below the 3rd percentile without increasing adverse outcomes.

## Resumo

**Objetivos** Avaliar o desempenho das curvas de Intergrowth-21 st (INT) e Fetal Medicine Foundation (FMF) na predição de resultados perinatais e de neurodesenvolvimento de recém-nascidos com peso abaixo do percentil 3.

**Métodos** Foram incluídas gestantes de feto único com idade inferior a 20 semanas de uma população geral em unidades de saúde não hospitalares. Seus filhos foram avaliados ao nascimento e no segundo ou terceiro anos de vida. Os recém-nascidos tiveram seus percentis de peso calculados para ambas as curvas. Sensibilidade, especificidade, valor preditivo positivo (VPP) e negativo (VPN) e área sob a curva ROC (ROC-AUC) foram calculados para desfechos perinatais e atraso de neurodesenvolvimento considerando o peso ao nascimento menor que o percentil 3 como ponto de corte.

**Resultados** Um total de 967 crianças foram avaliadas ao nascimento e no segundo ou terceiro anos de vida. A idade gestacional ao nascer foi de 39,3 (±3,6) semanas e o peso ao nascimento foi de 3.215,0 (±588,0) g. INT e FMF classificaram 19 (2,4%) e 49 (5,7%) recém-nascidos abaixo do percentil 3, respectivamente. A prevalência de parto pré-termo, intubação traqueal > 24 horas nos primeiros três meses de vida, Apgar de 5° minuto < 7, internação em unidade de terapia intensiva neonatal (internação em UTIN), taxa de cesariana e atraso de neurodesenvolvimento foi 9,3%, 3,3%, 1,3%, 5,9%, 38,9% e 7,3% respectivamente. Em geral, o percentil 3 de ambas as curvas apresentou baixa sensibilidade e VPP e alta especificidade e VPN. O percentil 3 de FMF mostrou sensibilidade superior para parto prematuro, internação em UTIN e taxa de cesariana. INT foi mais específico para todos os desfechos e apresentou maior VPP para o atraso do neurodesenvolvimento. Entretanto, exceto por uma pequena diferença na predição de parto pré-termo em favor de INT, as curvas ROC não mostraram diferenças na predição de resultados perinatais e de desenvolvimento neurológico.

**Conclusão** O peso ao nascer abaixo do percentil 3 segundo INT ou FMF isoladamente foi insuficiente para um bom desempenho diagnóstico de desfechos perinatais e de neurodesenvolvimento. As análises realizadas não puderam mostrar que uma curva é melhor que a outra em nossa população. INT pode ter vantagem em cenários de contingência de recursos, pois discrimina menos recém-nascidos abaixo do percentil 3 sem aumentar os desfechos adversos.

## Introduction

Fetal growth restriction is associated with adverse perinatal outcomes, neurodevelopmental delay, and the onset of chronic disease in adults. 1 2 3 Identification of fetuses and newborns (NB) with growth restriction could help improve these results by intensifying prenatal and postnatal care. 4 5

Several estimated fetal and birth weight (BW) charts have been published worldwide, showing significant differences. 6 7 8 9 10 11 12 While some authors suggest that these differences are due to racial and geographic variations, others attribute them to socioeconomic inequalities, nutritional deficits, or methods used in the studies. 7 9 13 14

One of the healthcare challenges in Brazil is to define which fetal and neonatal growth charts better discriminate children with growth restriction in the Brazilian population. It is unclear whether using North American or European curves could increase false positive diagnoses, as the cut-off points may be too high. Using references that underestimate diagnoses bears even more risk as it could deprive the most vulnerable pregnant women and their children of the necessary care, leading to an increase in the incidence of adverse outcomes.

Therefore, it should be understood that the random choice of a fetal or neonatal weight curve without an in-depth analysis of morbidity and mortality is not recommended. Choosing a particular reference over another is only justified if the reference can better identify the NB with the highest risk of morbidity and mortality without excess diagnoses. 15

The objective of this study was to evaluate the performance of two international BW curves, Intergrowth-21 st (INT) and Fetal Medicine Foundation (FMF) in predicting perinatal and neurodevelopmental outcomes in newborns based on birth weight below the 3rd percentile in a Brazilian city. This is the first prospective Brazilian study to include neurodevelopmental outcomes in assessing of birth weight curves.

## Methods

This prospective cohort evaluated children at birth and in the second or third years of life.

Data from a BRISA-RP cohort study were used. 16 The preliminary study assessed etiological factors of preterm birth and the consequences of perinatal factors on child health. The research ethics committee approved this study at the University Hospital where it was performed. Ribeirão Preto is located in south-eastern Brazil and has ∼710,000 inhabitants. This is a prosperous country region regarding income, consumption, and longevity. Birth data were collected between April 2010 and December 2011, and the neurodevelopmental assessment data in the second or third years of life.

Recruitment of this cohort started during pregnancy. Pregnant women from a general population with a single fetus aged less than 20 weeks were sequentially recruited from selected primary care units in this city. These units are part of the public health system, are not linked to the University, and generally serve a low- and middle-income population. The first ultrasound determined the gestational age (GA) used in this study. All pregnant women recruited had already undergone a first-trimester ultrasound with gestational age was calculated using the crown-rump length. Furthermore, all pregnant women underwent a new ultrasound to confirm the GA by certified physicians from the research team before 24 weeks. At birth, all NB with BW greater than or equal to 500 g were potentially eligible. NB older than 42 weeks were excluded to reduce the risk of bias in an incorrect recording of gestational age, as it is unlikely that a pregnancy will exceed this limit spontaneously or on medical advice. NB with severe malformations were also excluded. All cohort participants were encouraged to bring their children between the second and third years of life for a neurodevelopment assessment using the Bayley Scales of Infant Development, III edition (BSID-III). 17 All patients gave their informed written consent to participate in the study.

Sociodemographic and clinical data were collected, including maternal age, body mass index, race, parity, education level, smoking, alcohol use, hypertension, and diabetes. Data from the newborns of the included women were collected in their respective maternity hospitals in the city on birth or the following day by the research team.

The predictor variable was BW below the 3rd percentile for GA. The 3rd percentile was chosen because there is a high perinatal morbidity and mortality risk below this thresholds. 18 19 Furthermore, fetal or birth weight below the 3rd percentile is considered an isolated criterion for fetal and neonatal growth restriction, according to the latest expert consensus. 20 21

The perinatal outcomes were preterm birth, tracheal intubation for more than 24 hours in the first three months of life (Intubation), 5 minute Apgar <7, admission to a neonatal intensive care unit (NICU admission), and cesarean section rate. The long-term outcome variable was the risk of neurodevelopmental delay between the second and third years of life.

Percentiles of BW were obtained for each NB. INT (specific gender) and FMF calculators were used to predict BW. 22 23 These charts were chosen because both include fetal and neonatal charts and are the only ones whose calculators were found on official open access Web sites.

The INT standards were constructed from a prescriptive population of over 4,500 healthy pregnancies in a study of over 59,000 total pregnancies. The project involved 8 countries from 4 continents and included only highly selected women with optimal nutrition and low risk of pregnancy complications. Fetal anthropometric data were prospectively collected every 5 weeks starting at 14 weeks. The aim of that approach was to create charts that could be used worldwide. 9 24

Nicolaides et al. (FMF) used a heterogeneous sample of unselected pregnant women, most of them being white women from the United Kingdom. Data were collected from two sources. The first comprised 5163 paired measurements of EFW and BW, and the second of 95,579 pregnancies with EFW obtained by routine fetal ultrasound biometry. In this study, the authors proposed to consider that all babies of the same gestational age, even intrauterine babies, could be included for BW references. Thus, the construction of the curves considered that in a given population with a defined gestational age, the median fetal weight and the median birth weight are similar, with different degrees of deviations from the median for fetal weight and birth weight, depending on the gestational age. 10

Maternal, gestational, and childbirth data were obtained by filling out previously prepared questionnaires with interview data and medical records.

Neurodevelopment was assessed by ten psychologists who received identical, simultaneous, and group training. Children were assessed in three domains: cognitive, language (receptive and expressive), and motor (gross and fine), with each domain including specific tests for each age. For each age group (13–24 months; 25–42 months), there is a corresponding starting point. The child's performance on each test item was scored 0 or 1. The points obtained in each domain were summed, and children were classified as competent, emerging, or at risk according to the cut-off points provided by the test. A score that resulted in a risk for any domains was considered positive for neurodevelopmental risk.

There was no interference from researchers in prenatal care, labor and delivery, and postnatal care of children. All NB of pregnant women with conditions potentially associated with fetal growth deviations such as hypertension, diabetes, smoking, and preterm deliveries were included in the analysis.

Statistical analysis was performed using the SAS System for Windows (Statistical Analysis System), version 9.2., SAS Institute Inc, 2002–2008, Cary, NC, USA. Comparisons of descriptive variables were performed using the generalized estimating equations (GEE analysis) (numerical variables). Sensitivity, specificity, and positive and negative predictive values (PPV and NPV) of perinatal outcomes and risk of neurodevelopmental delay were estimated, with differences being determined by the McNemar test and GEE analysis considering a BW below the 3rd percentile as the cut-off point. The discriminatory ability of each curve was assessed using the AUC of the ROC curve. To be significant, were considered results with *p* -value <0.05 with a confidence interval of 95%.

## Results

### Participants

**Fig. 1:**
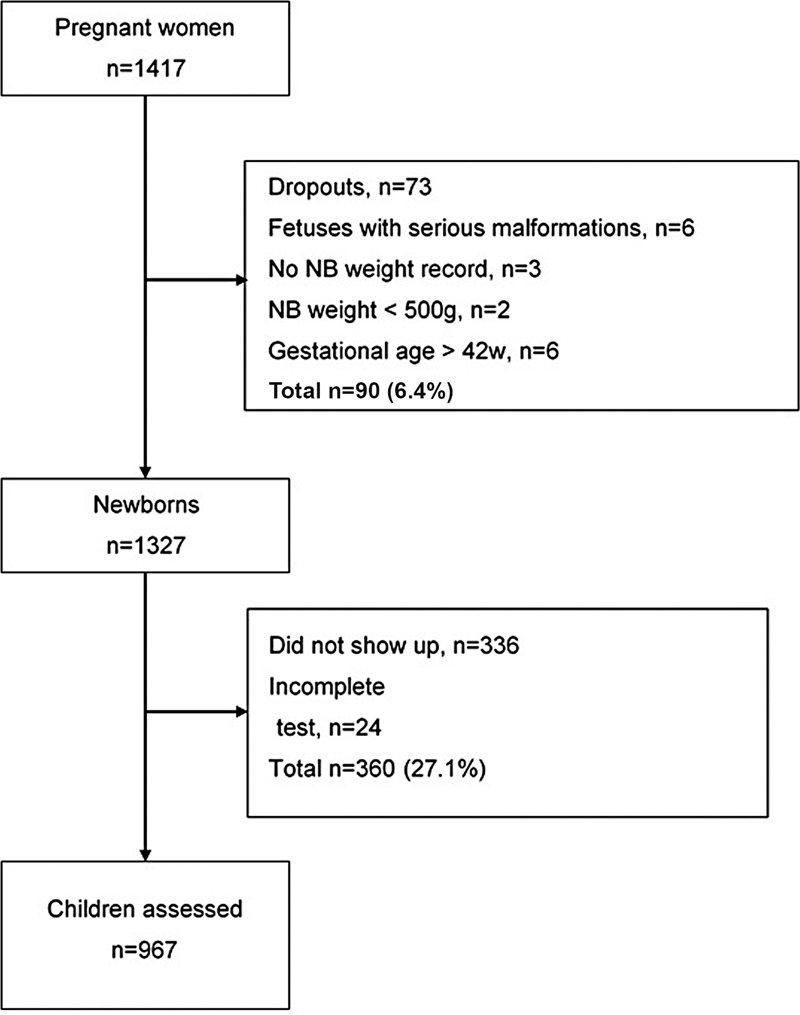
Flowchart of the participants included in the study.

A total of 1417 pregnant women were recruited. Seventy-three dropouts were reported, and 17 NB were excluded (6 with major malformations, 3 without weight records, 2 weighing less than 500 g, and 6 with a gestational age of 42 weeks or more). The total number of children with no or incomplete neurodevelopmental tests was 360 (27.1%). The final number of cases for analysis was 967 ( Fig. 1 ).

### Descriptive Data

The median age of pregnant women was 26.0 (±12.0) years. The majority (79.9%) were nuligest or secundigest, and 90.7% had full-term delivery. The median GA at birth, and the NB weight was 39.3 (±3.6) weeks, and 3,215.0 (±588.0) g respectively. The prevalence of hypertension was 15.4%, and smoking, 12.4%. The total prevalence of diabetes in the sample was 6.1%. In the INT and FMF groups, the diabetes rate was 10.5 and 4.1%, respectively. The overall prevalence of prematurity was 9.3%; however, among NB below the 3rd percentile, it was 46.2% for INT and 32.4% for FMF. For neurodevelopment, 741 children were evaluated between 13 and 24 months and 226 between 25 and 36 months ( Table 1 ). The prevalence of intubation, 5 minute Apgar <7, NICU admission, cesarean section and neurodevelopment delay was 3.3%, 1.3%, 5.9%, 38.9% and 7.3%, respectively ( Table 2 ).

**Table 1:** Cohort demographic characteristics

	Total n = 967	INT NB weight *p* < 3 n = 19	FMF NB weight *p* < 3 n = 49	p-value ^d^
Maternal age, years (IQR)	26.0 (12.0)	26.0 (12.0)	26.0 (12.0)	0.718
BMI, kg/m ^2^ (IQR)	26.5 (6.8)	27.8 (8.5)	27.0 (6.6)	0.218
Study US, weeks IQR)	22.9 (1.7)	23.1 (2.3)	22.9 (1.7)	0.919
Ethnicity				
- White (%)	510 (52.4)	11 (57.9)	27 (55.1)	0.756
- Non-white (%)	457 (47.3)	8 (42.1)	22 (44.9)	
Schooling				
- ≥12 years (%)	77 (8.0)	1 (5.3)	4 (8.2)	0.595
- <12 years (%)	890 (92.0)	18 (94.7)	45 (91.8)	
Marital status				
- Married or cohabiting (%)	778 (80.5)	14 (73.7)	37 (75.5)	0.810
- No partner (%)	189 (19.5)	5 (26.3)	12 (24.5)	
Parity				
- 0–1 (%)	773 (79.9)	16 (84.2)	43 (87.8)	0.515
- ≥2 (%)	194 (19.5)	3 (15.8)	6 (12.2)	
NB sex				
- Male (%)	474(49.0)	11 (57.9)	23 (46.9)	0.228
- Female (%)	493 (51.9)	8 (42.1)	26 (53.1)	
Hypertension a (%)	149 (15.4)	4 (21.1)	12 (24.5)	0.667
Diabetes a (%)	59 (6.1)	2 (10.5)	2 (4.1)	<0.001
Smoking ^b^ (%)	120 (12.4)	6 (31.6)	11 (22.5)	0.189
Alcohol ^b^ (%)	238 (24.6)	6 (31.6)	14 (28.6)	0.706
Delivery, weeks (IQR)	39.3 (3.6)	39.6 (4.6)	39.3 (3.4)	0.220
≥37 weeks	39.4 (1.7) ^e^	40.0 (1.6) ^g^	39.7 (1.4) ^i^	
< 37 weeks	35.7 (2.4) ^f^	35.9 (1.0) ^h^	34.8 (2.3) ^j^	
NB weight, g (IQR)	3215.0 (588.0)	2425.0 (660.0)	2455.0 (465.0)	0.464
≥37 weeks	3250.0 (570) ^e^	2500.0 (135) ^g^	2500.0 (295) ^i^	
< 37 weeks	2645.0 (877.5) ^f^	1842.5 (178.8) ^h^	1735.0 (381.3) ^j^	
Breastfeeding ≥1month (%)	863 (89.3)	18 (94.7)	41 (83.7)	0.187
Day care center (%)	469 (48.5)	4 (21.1)	12 (24.5)	0.667
Neurodevelop. Assessm ^c^ , years (IQR)	1.9 (0.3)	1.8 (0.3)	1.8 (0.3)	0.428

Abbreviations: BMI, body mass index; FMF, Fetal Medicine Foundation; INT, Intergrowth-21st; IQR, interquartile interval; NB, newborn; p, percentile; US, ultrasound.

a Types of hypertension included chronic, gestational, or preeclampsia. Types of diabetes included: type 1, 2 our gestational; ^b^ Any amount of consumption; ^c^ Child's age at evaluation of neurodevelopment by Bayley III test; ^d^ Comparisons of categorical and numerical variables considering only newborns with <p3 in each curve, through GEE (Generalized Estimating Equations) analysis; ^e^ n = 877; ^f^ n = 90; ^g^ n = 13; ^h^ n = 6; ^i^ n = 37; ^j^ n = 12.

**Table 2:** Performance of birth weight below the 3rd percentile for perinatal, and infant neurodevelopment outcomes according to Intergrowth-21 st and fetal medicine foundation curves

		n (%)	Sensitivity% (95%CI)	p-value a	Specificity% (95%CI)	p-value a	PPV (95%CI)	p-value ^b^	NPV (95% CI)	p-value ^b^
Preterm birth n = 90 (9.3%)	INT	6 (31.6%)	6.7 (2.7–14.5)	0.014	98.5 (97.4–99.2)	<0.001	31.6 (13.6–56.5)	0.334	91.1 (89.1–92.8)	0.136
	FMF	12 (24.5%)	13.3 (7.4–22.5)		95.8 (94.2–97.0)		24.5 (13.8–39.2)		91.5 (89.5–93.2)	
Intubation n = 32 (3.3%)	INT	2 (10.5%)	6.3 (1.1–22.2)	0.083	98.2 (97.0–98.9)	<0.001	10.5 (1.8–34.5)	0.952	96.8 (95.5–97.8)	0.217
	FMF	5 (10.2%)	15.6 (5.9–33.6)		95.3 (93.7–96.5)		10.2 (3.8–23.0)		97.1 (95.7–98.0)	
5 minute Apgar <7 n = 13 (1.3%)	INT	0	0.0 (0.0–28.4)	0.317	98.0 (96.8–98.8)	<0.001	0.0 (0.0–20.9)	1.00	98.6 (97.6–99.2)	0.537
	FMF	1 (2.0%)	7.7 (0.4–37.9)		95.1 (93.4–96.3)		2.1 (0.1–12.5)		98.7 (97.7–99.3)	
NICU admission n = 58 (5.9%)	INT	2 (10.5%)	3.5 (0.6–13.0)	0.014	98.1 (97.0–98.9)	<0.001	10.5 (1.8–34.5)	0.429	94.1 (92.4–95.5)	0.065
	FMF	8 (16.3%)	13.8 (6.6–25.9)		95.5 (93.9–96.7)		16.3 (7.8–30.2)		94.6 (92.8–95.9)	
Cesarean n = 376 (38.9%)	INT	8 (42.1%)	2.1 (1.0–4.3)	<0.001	98.1 (96.6–99.0)	<0.001	42.1 (21.1–66.0)	0.327	61.2 (58.0–64.3)	0.060
	FMF	17 (34.7%)	6.7 (4.4–9.8)		95.9 (93.9–97.3)		51.0 (36.5–65.4)		61.8 (58.5–64.9)	
Neurodevelopment n = 71 (7.3%)	INT	4 (21.1%)	5.6 (1.8–14.5)	0.317	98.3 (97.2–99.0)	<0.001	21.1 (7.0–46.1)	0.005	92.9 (91.1–94.4)	0.257
	FMF	5 (10.2%)	7.0 (2.6–16.4)		95.1 (93.4–96.4)		10.2 (3.8–23.0)		92.8 (90.9–94.4)	

Abbreviations: FMF, Fetal Medicine Foundation ( *n* = 49); INT, Intergrowth-21 ^st^ ( *n* = 19); Intubation, tracheal intubation for more than 24 hours in the first three months of life; Neurodevelopment, neurodevelopment delay (assessment in the second and third years of life by the BayleyIII test); NICU, neonatal intensive care unit; VPN, negative predictive value; VPP, positive predictive value.

a Mc Nemar test, ^b^ GEE (generalized estimating equations).

## Main Results

**Fig. 2:**
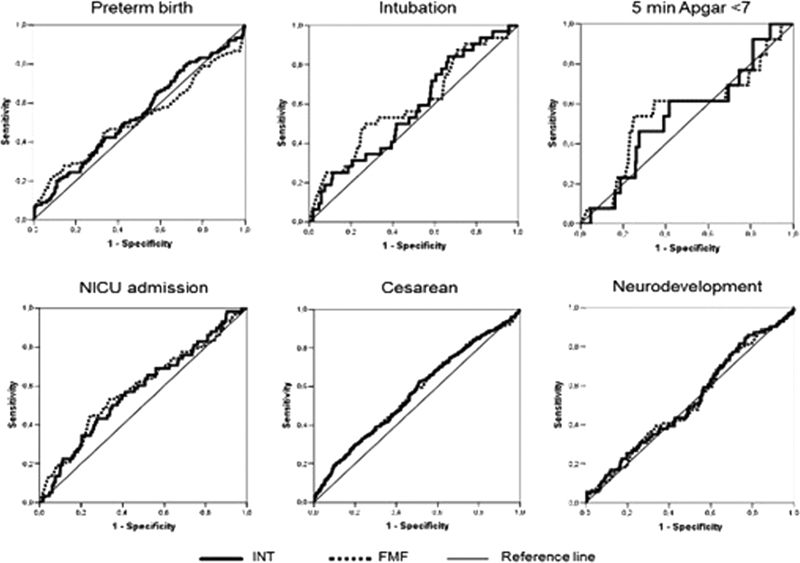
Receiver operating characteristic curve of Intergrowth-21 st and Fetal Medicine Foundation to predict perinatal and infant neurodevelopmental outcomes using birth weight percentiles.

INT and FMF classified 19 (1.9%) and 49 (5.1%) NB below the 3rd percentile, respectively. As a rule, high specificity was observed, but low sensitivity and positive predictive value. Some results were statistically superior for FMF, such as sensitivity for preterm delivery, NICU admission, and cesarean, respectively 13.3% (95%CI 7.4–22.5) versus 6.7% (95%CI 2.7–15.5) ( *p* =.014), 13.8% (95%CI 6.6–22.9) versus 3.5% (95%CI 0.6–13.0) ( *p* = .014) and 6.7% (95%CI 4.4–9.8) versus 2.1% (95%CI 1.0–4.3) ( *p* < .001). On the other hand, INT was superior in all outcomes when specificity was evaluated ( *p* < .001). In addition, the NPV for neurodevelopment delay was 21.1% (95%CI 7.0–46.1) for INT versus 10.2% (95% CI 3.8–23.0) for FMF ( *p* = .005) ( Table 3 ). However, analysis using ROC curves did not show adequate performance in predicting perinatal and neurodevelopmental outcomes ( Fig. 2 ).

**Table 3:** Comparison of area under receptor operating characteristics curve for prediction of perinatal and infant neurodevelopmental outcomes between intergrowth-21 st and fetal medicine foundation using birth weight percentiles

	INT	FMF	p-value
	ROC curve AUC (95%CI)	ROC curve AUC (95%CI)	
Preterm birth	0.54 (0.47–0.60)	0.52 (0.45–0.59)	0.032
Intubation	0.57 (0.48–0.67)	0.60 (0.49–0.70)	0.090
5 minute Apgar <7	0,54 (0.39–0.70)	0.56 (0.39–0.73)	0.437
NICU admission	0.58 (0.50–0.66)	0.59 (0.51–0.67)	0.319
Cesarean	0.57 (0.53–0.60)	0.56 (0.53–0.60)	0.887
Neurodevelopment	0.52 (0.45–0.59)	0.52 (0.45–0.59)	0.453

Abbreviations: FMF, Fetal Medicine Foundation; INT, Intergrowth-21st; Intubation, tracheal intubation for more than 24 hours in the first three months of life; Neurodevelopment, neurodevelopment delay (assessment in the second and third years of life by the BayleyIII test); NICU, neonatal intensive care unit; ROC curve AUC, area under receptor operating characteristics curve.

## Discussion

For both curves evaluated, the cutoff point for birth weight below the **3rd percentile** alone did not prove to be a good predictor of adverse perinatal outcomes and risk of neurodevelopmental delay. FMF classified more than double NB with BW below the 3rd percentile. This increased the sensitivity but did not improve the other parameters.

The sample size was one of the limitations of the study because some outcomes such as intubation ( *n* = 32), 5 minute Apgar score <7 ( *n* = 13), and NICU admission ( *n* = 58) had a low incidence, resulting in lower reliability of the results. Until birth, few cases were excluded or dropped out of the study ( *n* = 90; 6.4%); however, many children did not attend the neurodevelopment test ( *n* = 360; 27.1%). We were unable to investigate the reasons for the withdrawal of pregnant women and the absence of children. Data on the quality of management of fetal growth restriction pregnancies, perinatal deaths, the onset of diseases, and quality of infant stimulation are scarce. The high number of absences may cause some bias in the results, as perinatal losses usually occur among those with the lowest weight percentile, which would probably influence the INT group more because it concentrates the smallest NBs. However, it is essential to remember that absences interfere in analyzing both patterns since the study group is the same.

It is important to note that this study only included newborns weighing below the 3rd percentile. We did not include the restricted fetuses because we did not have estimated fetal weight and Doppler data. Therefore, probably some NBs above the 3rd percentile but at risk were not included.

The prospective design add advantages to the study, as it was possible to obtain a sample of pregnant women of non-hospital origin and were still in the first half of pregnancy. Including pregnant women with complications such as hypertension and smoking was purposeful, as low birth weight can result from multiple maternal and gestational conditions. Thus, we intended to obtain a sample that well represented the general population with its proportion of healthy women and others with prevalent diseases during pregnancy.

The main strength of this study was the inclusion of neurodevelopmental outcomes, as the predictive capacity of perinatal outcomes is low. 25 26 Long-term results are essential to assess the role of gestational complications such as low birth weight in the onset of permanent neurological damage, which is difficult to assess in the neonatal period. 27 This is the first prospective Brazilian study to include neurodevelopmental outcomes in assessing birth weight curves.

We believe that the assessment of the curves made in this study was timely, as they represent two distinct types of population samples. One of them (INT) is based on intercontinental and multi-ethnic populations, including the Brazilian population, while the other (FMF) is predominantly based on the population of European women. 9 10

Both curves were poor predictors of perinatal and neurodevelopmental outcomes, probably because these outcomes are influenced by multiple factors beyond the birth weight, such as prematurity, birth conditions, neonatal care, breastfeeding, and infant stimulation. This has already been demonstrated in other studies with fetal and neonatal patterns. 28 29 The most remarkable difference between the curves in this study was the highest cut-off point on the FMF graph. INT discriminated 19 NB below the 3rd percentile while FMF 49. In practice, we had more than twice as many newborns classified as having restricted growth according to the new consensus for one standard (FMF) compared with the other (INT). To exemplify, if we had a hypothetical 39-week NB weighing 2,580 g, this NB would be in the fifth (male chart) or seventh (female chart) percentiles of the INT, but by FMF references, it would be in the second percentile. This difference may seem insignificant, but it can place many additional newborns in the growth-restricted group in a population context. Changes in cutoff points could make any growth curve potentially suitable for any population. We could have tested other thresholds to define the one that best discriminates the children at the most significant risk in each pattern. 30 However, we did not do this because it would be of little practical use as most hospitals have their chosen fetal and neonatal growth reference charts and well-known thresholds such as the 3rd or 10th percentile. Some expected differences were found with the applied statistical tests due to differences in the cutoff points of the evaluated curves. Higher sensitivity for FMF and higher specificity for INT. However, the ROC curves, except for a small difference in the prediction of preterm birth in favor of INT, did not show consistent differences.

The birth weight of our sample was similar to that of other national studies. Barros et al. conducted a study in Pelotas, a city in southern Brazil, with a cohort of 4,558 newborns. Most pregnant women came from urban areas, and 61.7% were white. The BW was 3,149.6 g, slightly lower than in this study (3,215.0 g). However, the sample by Barros et al. included a higher proportion of pregnancies with complications such as smoking (27.5%), premature birth (15.3%), and hypertension (23.7%). 31 Kiserud et al., similarly to the INT project, used a multinational sample that included a Brazilian city (Campinas; *n* = 150) to create EFW and BW standards for the World Health Organization. 7 Interestingly, the GA at the birth of the Brazilian sample was 39 weeks, similar to this study (39.3 weeks), and the BW was 3290.0 g, also similar to this study (3243.0 g) when growth-restricted NB are excluded.

The prevalence of premature births was 9.3%, lower than that found in the study by Passini et al. (12.3%). 32 The study cited above included 20 referral hospitals and more than 33,000 deliveries in Brazil. This difference can be explained by the characteristics of the samples obtained in each study. Our study recruited pregnant women in non-hospital units, while the study by Passini et al. included pregnant women from referral hospitals. Although some of the pregnant women in our study had complications during pregnancy, pregnant women coming from referral hospitals are more likely to have risk factors for preterm delivery, whether spontaneous, due to rupture of membranes, or therapeutic. In addition, the study by Passini et al. showed that Brazilian regions presented slightly different prevalences, with a lower prevalence for the Southeast region, where our study was performed.

Among the maternal diseases that can negatively affect fetal growth and bias the analyses, arterial hypertension had a slightly higher prevalence in this study (15.2%) compared with the general population. 20 33 We postulate that the recruitment may have been biased because the hypertensive pregnant woman seeks the health unit more frequently. The prevalence of smoking was similar to other studies. 34 The prevalence of diabetes was low in this study, despite the indistinct inclusion of type 1, 2, and gestational diabetes, probably due to underreporting and the use of old diagnostic criteria for gestational diabetes used at the time. The International Association of Diabetes in Pregnancy Study Group criteria, which are more sensitive, were adopted from 2015 onwards in our country. The statistical difference observed between the INT and FMF groups is probably due to the small number of diabetes diagnoses in these groups (the same 2 cases for both groups).

The INT and FMF curves were tested in different populations. Kajdy et al., in Poland, obtained a BW reference curve of 39,092 single births and compared their percentiles with 6 published charts, including the INT. In that study, the 50th percentile at 40 weeks was 3645.8 g and 3486.7 g for male and female NB, respectively. The authors obtained 3.2% of NB below the 3rd percentile by the local chart and only 0.6% by INT. 35 Anderson et al. obtained data from 53,484 NB in Auckland, New Zealand, and compared small-for-gestational-age (SGA) new-born outcomes between INT versus a customised standard using maternal characteristics of height, weight, parity, and ethnicity. The GA was 39.4 weeks, and the weight was 3433 g, with a higher weight associated with Pacific ethnicity (3585 g) and a lower weight associated with Indian ethnicity (3130 g). The incidence of SGA was 4.5% when INT was used and 11.6% when the customised standard was used. The authors concluded that customised curves identified more NB SGA at risk for perinatal morbidity and mortality than INT standards. 36 Francis et al. analyzed data from 1.25 million full-term pregnancies from 10 countries. INT was compared with a customised standard to determine stillbirth rates in SGA and large for gestational age (LGA) groups. Significant differences in SGA rates would be found between countries using INT. The most significant differences were observed between Sweden (10.7% for the customised standard and 3.1% for INT) and India (11.3% for the customised standard and 16.8% for INT). In Sweden ( *n* = 257,924), the GA at birth was 40.8 weeks, and the BW was 3623.0 g, while in India ( *n* = 6436) the GA at birth was 39.0 weeks, and the BW was 3055.5 g. 37

Regarding FMF patterns, Duncan et al. compared the detection of SGA in preterm prelabor rupture of membranes by Hadlock versus the FMF charts. A sample of 106 patients from a university hospital in Tennessee with 84.9% African American was assessed. The cutoff point adopted was the 10th percentile. In this study, the FMF and Hadlock patterns discriminated respectively 48 (45%) and 22 (21%) of NB below the 10th percentile. Both patterns had similar accuracy in predicting SGA and were equally poor in predicting severe adverse neonatal outcomes. The FMF chart resulted in a 2-fold increase in positive cases, potentially increasing surveillance. 38

Based on the studies cited above, it is evident that different populations can provide different proportions of NB below the 3rd or 10th percentile when using the same curve. Given its miscegenation, it is plausible that the INT provides standards more suited to the Brazilian population as the multiethnic sample is one of the main features of the INT standards. Compared with FMF, INT is less sensitive but appears safe as it does not increase adverse outcomes. This can be advantageous in resource contingency scenarios.

## Conclusion

Although BW below the 3rd percentile is associated with adverse perinatal and neurodevelopmental outcomes, it was insufficient for a good diagnostic performance when evaluated alone. The analyses performed in this study could not show that one curve is unequivocally better than the other in our population. The apparent excess of newborns classified below the 3rd percentile by FMF may mean that it is not advisable to use references imported from countries of different racial composition, such as European countries or the United States. It is plausible that INT is more suitable for the Brazilian population due to its mixed racial composition. Further prospective studies are needed in Brazil to compare global standards, such as INT, with locally customized curves.
